# Influence of Microgroove Structure on Cutting Performance and Chip Morphology during the Turning of Superalloy Inconel 718

**DOI:** 10.3390/ma14154142

**Published:** 2021-07-25

**Authors:** Zhongfei Zou, Lin He, Hongwan Jiang, Sen Yuan, Zhongwei Ren

**Affiliations:** 1 School of Mechanical Engineering, Guizhou Institute of Technology, Guiyang 550003, China; hwjiang@git.edu.cn (H.J.); syuan@git.edu.cn (S.Y.); renzw@git.edu.cn (Z.R.); 2 School of Mines and Civil Engineering, Liupanshui Normal College, Liupanshui 553004, China; helin6568@163.com; 3 School of Mechanical Engineering, Guizhou University, Guiyang 550025, China

**Keywords:** microgroove, Inconel 718, cutting temperature, tool life, chip morphology

## Abstract

This study designed a new microgroove cutting tool to machine Inconel 718 and focused on the effect of microgroove structure on the cutting performance and chip morphology during the turning. A comparative analysis of the cutting force, cutting temperature, tool life, tool wear, and chip morphology of the microgroove cutting tool and the original cutting tool was conducted. The main cutting force and temperature of the microgroove cutting tool were reduced by 12% and 12.17%, respectively, compared with the original cutting tool. The microgroove cutting tool exhibited a significant improvement compared with the original cutting tool, which extended the tool life by up to 23.08%. Further, the microgroove cutting tool distorted the curl radius of the chips extensively. The experimental results showed that the microgroove structure can not only improve the tool life, but also improve the chip breaking effect.

## 1. Introduction

Superalloys are important, irreplaceable aeroengine materials that are used in the modern aviation industry. The superalloy material type is based on iron, nickel, and cobalt and can work under a high temperature and pressure for an extended period. Among them, nickel-based superalloys have a good oxidation resistance, high temperature strength, corrosion resistance, fatigue property, and fracture toughness [1]. However, because of the difficult machining characteristics of the superalloys, a severe thermal mechanical load is generated at the tool–chip interface on the rake face of the cutting tool, which results in an accelerated wear and reduced tool life.

To solve the abovementioned problems, oil-based fluid is used commonly during the turning of Inconel 718. Although the cooling fluid can reduce the temperature and remove a large amount of heat from the tool–chip contact zone, the cost of recycling and the disposal of cutting fluid is expensive, and strict environmental protection policies that are stipulated in the Occupational Safety and Health Act (OSHA) must be observed [2]. Because of these factors, the development of lubrication conditions, surface texturing, improvements in tool geometry, and the application of coatings has been suggested by researchers to solve the abovementioned problems [3,4,5].

For instance, many scholars have used different lubrication methods to improve the cutting performance and the tool life. Yıldırım et al. [6] evaluated the cutting temperature at the tool–chip interface, surface roughness, and tool wear to machine Inconel 625 in four different cutting environments, such as dry, MQL, cryogenic, and nanofluids. Results indicated that a 0.5 vol% hexagonal boron nitride cooling method combined with liquid nitrogen has the best turning performance. Necati and Cicek [7] investigated the drilling performance of Inconel 718 in different drilling environments. A better surface finish and tool life was obtained during drilling in wet and cryogenic environments compared with a dry environment. Ucan et al. [8] experimentally studied the micromilling of Inconel 718 under cryogenic precooling, MQL, and dry machining environments. Authors reported that cryogenic precooling of a workpiece reduced the surface roughness and burr formation but yielded a poor tool life in cryogenic precooling compared with MQL and dry conditions. Pusavec et al. [9] investigated the sustainability of dry, near-dry (MQL), cryogenic, and cryo + MQL processes to turn Inconel 718 compared with the traditional processing technique; the results showed that cryo + MQL reduces the feed force up to 50%. Abbas et al. [10] studied the effect of three environmentally friendly cooling strategies on surface quality and power consumption in finishing end milling. Results indicated that the effectiveness of lubricant with nanoparticles in reducing the friction and thermal damages on machined surface when machining with MQL was comparable with the case of MQL + Al_2_O_3_. Zhang et al. [11] proposed a new hybrid process that was termed MQCL and found that the tool life increased 1.5 times, the cutting force was reduced because of a low coefficient of friction in the cutting zones, and the sustainability of the milling of Inconel 718 improved compared with the dry-milling condition. Therefore, it can be concluded from the above literature that the small cooling/lubrication fluids (CLFs) quantities method is an effective alternative for solving the problem of high cutting temperature and short tool life in the cutting process.

In addition, to improve cutting performance, the geometric shape of the tool has been continually improved, especially the microtexturing technology. Some scholars found that microtexturing technology resolved the traditional cutting problems, especially for materials that are difficult to process. The performance of nano/microgrooves with various geometric shapes on the rake or flank face of cutting tools has been investigated, such as a vertical [12], parallel [13], cross [14], elliptical [15], dot, and pit [16,17] texture tools. The appearance of microtexturing technology enhanced the friction performance of the friction pair surface and decreased the surface wear and surface-bearing capacity [18,19]. Pang et al. [20] designed three geometrically shaped microtexture drilling tools and compared their cutting performance. They concluded that the use of microtextured drilling tools to machine Inconel 718 decreases the cutting temperature (by ~4.6% to 8.9%), thrust force, and tool wear; improves the surface quality; and extends the cutting tool service life. Nageswaran et al. [21] found that the combination of a cylindrical dimple on the rake face and the square pyramid texture on the flank face improved the wear resistance of the tool, and the tool durability improved by approximately one third relative to the conventional tool. Munish et al. [22] compared the performance of textured and non-textured tools under three experimental conditions. Nanofluid with texturing tools yielded excellent results compared with other cooling situations. Zhou et al. [23] studied the coupling effects of microtextures and nanofluids experimentally, based on the cutting performance of cutting tools in different cutting situations. The cutting force, surface roughness, and tool wear rate in the TP (textured with micro-groove parallel) + NC (nanofluids) cutting situation decreased by 38.4%, 27.75%, and 63.3%, respectively, compared with those of NT (non-texture) + CC (conditional cutting fluid). Li et al. [24] proposed a grinding method to manufacture microgroove texturing on the rake face of cutting tools using a diamond grinding wheel and found that the cutting forces that were caused by the microgroove inserts decreased. The microgroove insert showed a better wear resistance compared with the conventional insert, and no chipping resulted. Wu et al. [25] experimentally studied the tribological performance of micro-groove tools in turning AISI 304 process. They concluded that compared with the conventional tool, the main cutting force, the feed resistance, and the cutting depth resistance of the proposed micro-groove tool were reduced by 16.1%, 33.9%, and 40.1%, respectively. In addition, the cutting temperature was reduced by 17.2% and the wear width of the flank face was reduced by 36.7%. Pang et al. [26] produced symmetrical conical microgrooved texturing on the rake face of the cemented carbide by laser texturing. The texture improved the cooling and lubrication effect of the cemented carbide and reduced the friction coefficient at the tool–chip contact zone.

These previous studies mainly found that a microtexture tool is useful for improving tool life. However, some researchers reported that microtexture tools exert an important influence on chip morphology. For example, Munish et al. [22] indicated the chip morphology was shorter, with curved and short-radius chips that were formed by the texturing tools. Guo et al. [27] found that the curling degree of the chips can be changed by the tool with microgroove textures that were manufactured on the rake face, the contact length at the tool–chip interface was shortened, and the chip breaking ability was improved. The microtextures enhanced the internal cooling abilities, and, thus, the cutting force was reduced and the adhesive resistance and abrasive wear resistance of the material improved. Darshan et al. [28] pointed out that the microchannel textured inserts played the role of chip breaker, which decreased the contact length between the tool and chip, paving the way for a more rational chip shape. The microchannel texture achieved certain functional tasks, which were to decrease the chip curvature radius, plastically deform the chips, and break chips into short pieces. Zheng et al. [29] emphasized that compared with non-textured tools, the chips that were formed by textured tools had a weaker deformation. The texture controlled the formation, shape, and size of the chips, which completed the chip breaking and curling procedure, and improved the cutting performance significantly. Furthermore, cooling mode also has an important influence on chip formation. Maruda et al. [30] emphasized the influence of the cooling conditions on the cutting tool wear, the chip shape, and the indices of the chip formation zone. The study of chip formation shows desirable chip shapes with application of the MQCL method with phosphate esterbased additive, which facilitates chip removal from the cutting zone. Setti et al. [31] have proved that effective lubrication with nanofluid results in formation of chip which reduces the tool wear of the flat area on wheel surfaces. Therefore, the influence of new lubrication and cooling methods or new microstructure of tool on chip formation and morphology is a future research direction.

The research described above improved the cutting performance indirectly by controlling the friction effect of the tool–chip interface. The substantial improvement is attributed to several physical mechanisms, such as tool–chip contact length reduction, hydrodynamic effects, and lubricant storage in microcavities to improve the load-bearing capacity. However, the size and distribution of the microtexture in the aforementioned studies [12,13,14,15,16,17] were initially set over an empirically established range, and thus the final structures were determined in coordination with orthogonal experimental results. In other words, the method of determining the optimal shape and size of a microtexture has no specific design theory basis. Therefore, in previous work [32,33], we designed several microgroove tools that turn hard-to-machine materials based on temperature field contours. Our innovative design for a microgroove cutting tool was introduced, and abrasive, adhesive, and oxidation wear were found to be the dominant wear mechanisms. In addition, the experimental results showed that the microgroove tools designed in our previous work had excellent wear resistance relative to original tools. We note that in our previous study, we did not discuss the influence of the microgroove tool on chip morphology and chip breaking, nor the effect of chip burr on tool wear. Therefore, the main purpose of this current study was to develop a microgroove tool for turning Inconel 718 according to our previous design method, as well as analyze and compare the cutting performance of the tool and chip morphology with the original cutting tool and microgroove cutting tool during the turning of superalloy Inconel 718. The analysis included the cutting temperature, cutting force, tool wear behavior, tool life, and chip morphology. The proposed microgroove cutting tool demonstrates excellent wear resistance and a superior lifetime, and the microgroove cutting tool is shown to have a good chip breaking effect.

## 2. Materials and Methods

### 2.1. Experimental Tests

**Figure 1:**
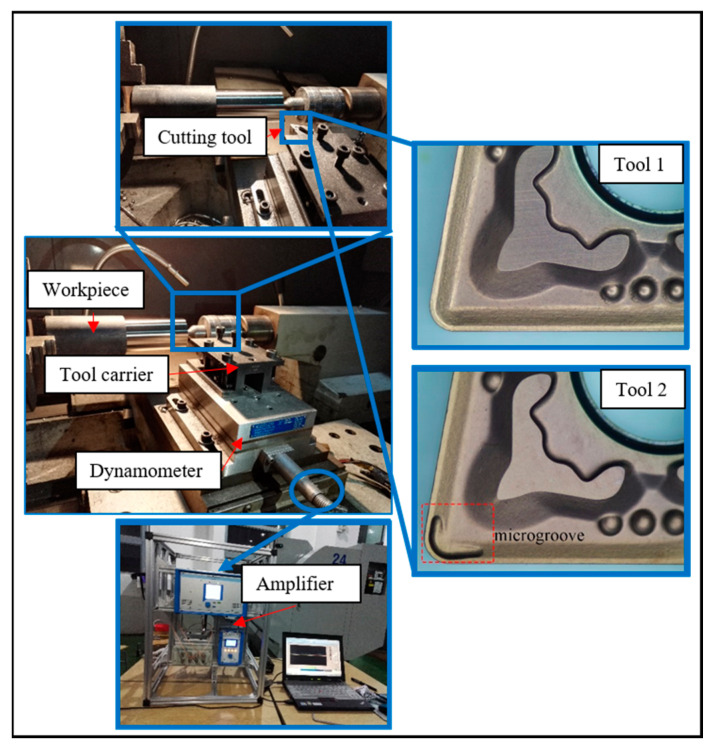
Experimental setup of turning superalloy Inconel 718 and cutting tools.

Two cutting tools were supplied by Zigong Cemented Carbide Co., Ltd., Zigong, China, and were made from WC that is commonly used to machine Inconel 718. The two tools included the original cutting tool (Tool 1), which was designed and provided by the manufacturer, and a second microgroove cutting tool (Tool 2), which was an improved design from our previous study [30]; both tools are shown in Figure 1. The microgrooves were scored 1.6 mm in length and 1.3 mm in width, and the depth was 0.12 mm. The two tools used the same PVD TiAlN coating (4 μm, Ti: 35.66 wt%, Al: 31.98 wt%, N: 32.26 wt%) and powder metallurgy compaction process. The composition of the tool substrate material was M30 (W: 84.8 wt%, Co: 9.1 wt%, C: 6.1 wt%). The tool geometrical parameters of both tools were equivalent, such as a relief angle of 5°, a rake angle of 8°, and an inclination angle of 7°. The tools were clamped in a DCLNR 2020K12 tool carrier with an approach angle of 95°.

Cutting experiments were performed on a C2-6136HK CNC lathe with coolant, as shown in Figure 1. The detailed input process parameters and responses that were used in the study are listed in Table 1. The recommended cutting parameters from the tool manufacturer were a cutting speed of $v_{c}=35\;m/\min$, depth of cut $a_{p}=1.5\;mm$, and feed rate f=0.1 mm/rev. The workpiece was Inconel 718 cylindrical bar through solid solution and aging heat treatment process, with the chemical composition and physical parameters as shown in Table 1 and Table 2, respectively.

During machining, a fresh cutting edge was used for each group test, and the cutting force measurement was repeated three times to minimize errors in the results. We used the KISTLER 9257B dynamometer to measure the cutting forces in real time during the turning of the Inconel 718. When each cutting test group was completed, the chips were collected and observed using an Olympus BX51-P optical microscopy. A Zeiss SUPRA 40 scanning electron microscope was used to obtain scanning electron microscopy (SEM) and energy dispersive spectroscopy (EDS) spectra of the chips.

To compare the durability of the two tools, we carried out a tool life test using the recommended parameters. The workpiece diameters were 60 mm. During testing, Tools 1 and 2 were cleaned ultrasonically at 3-min intervals, and the tool wear of the rake and flank surfaces was detected by an Olympus BX51-P optical microscope. The cumulative flank wear as measured for each tool was recorded at each interval. We set the maximum wear standard (VB) to 0.2 mm on the flank face. The three orthogonal components of the cutting force during cutting were measured by dynamometer.

### 2.2. FEM Simulations

**Figure 2:**
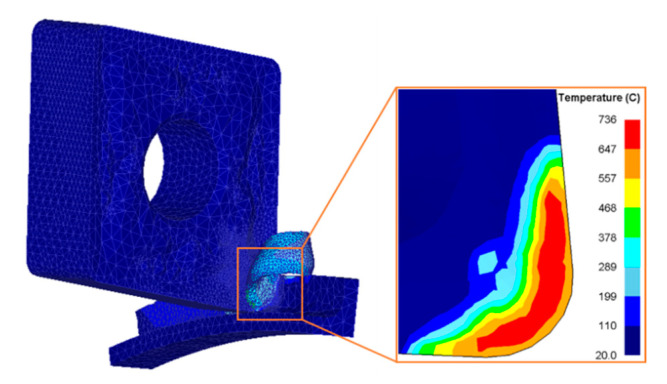
Simulation result showing surface temperature of Tool 1 in stable temperature situation.

The workpiece was fixed, and the tool was rotated around the workpiece axis. The parameter settings for the finite-element simulation are reported in Table 3. The cutting simulation finished at the 3598th step. During the cutting simulation, the cutting tool temperature increased gradually, and it tended to be stable when it exceeded 0.01 ms. Figure 2 shows the simulation result of Tool 1 when the cutting temperature stabilized.

## 3. Results and Discussion

### 3.1. Effect of Microgroove on Cutting Force and Temperature during the Turning of Superalloy Inconel 718

The cutting force and temperature are significant factors in the evaluation of cutting tool performance, because they affect the tool life directly. The cutting force and temperature of the two tools from the cutting experiment and simulation were compared, and the cutting performance of both turning tools was analyzed.

**Figure 3:**
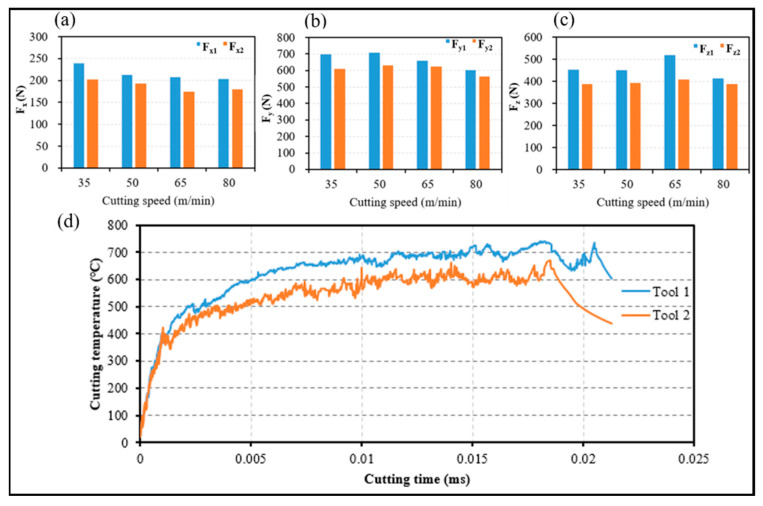
Comparison of cutting forces and cutting temperature for two tools: (**a**) the cutting depth resistance, (**b**) the feed resistance, (**c**) the main cutting force, (**d**) cutting temperature.

The cutting forces at different cutting speeds when the two tools were used to machine the workpiece materials are shown in Figure 3a–c, and the simulation results of the cutting temperatures for both tools are plotted in Figure 3d.

The cutting force of Tool 1 is larger than that of Tool 2 at different cutting speeds from Figure 3a–c. For the recommended cutting parameters, the cutting depth resistance $F_{x}$ and the feed resistance $F_{z}$ of Tool 2 decreased by 15% and 14%, respectively, relative to those of Tool 1. However, the main cutting force $F_{y}$ decreased by 12%. Figure 3d shows that during the steady state turning of Inconel 718, the average cutting temperature of Tool 1 was 12.67% greater than that of Tool 2.

**Figure 4:**
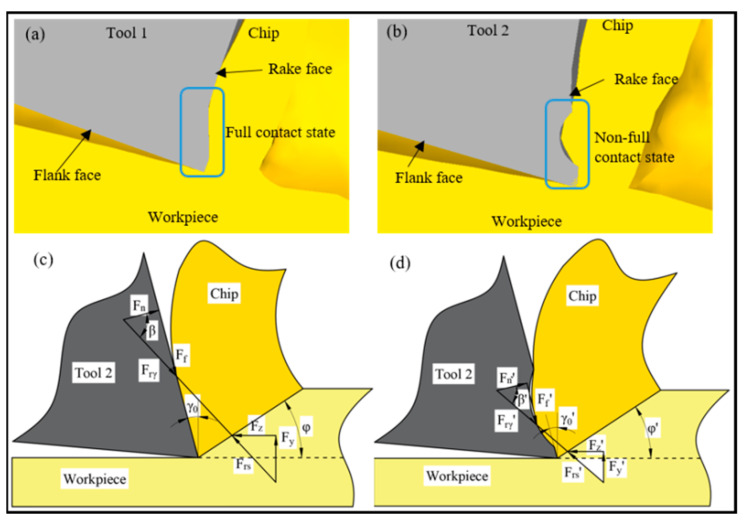
Comparison of tool–chip contact and force balance relationships for two tools: (**a**) the tool–chip contact of Tool 1, (**b**) the tool–chip contact of Tool 2, (**c**) the force balance relationship of Tool 1, (**d**) the force balance relationship of Tool 2.

We concluded that the cutting forces and temperatures decreased after use of the microgroove cutting tool because the microgroove design and implementation changed the primary contact form of the tool–chip. The tool–chip contact form of Tool 1 was full contact, whereas that of Tool 2 was not full contact, as shown in Figure 4a,b. Based on our previous research [28], the force-balance relationships in Figure 4c,d for the two tools are given as:

(1) $$F_{n}=F_{r\gamma }\cdot \cos\beta$$

(2) $$F_{x}=F_{r\gamma }\cdot \sin(\beta +\gamma_{0})$$

(3) $$F_{y}={\left(F_{r\gamma }{}^{2}-F_{x}{}^{2}\right)}^{1/2}$$

where, $F_{x}$ and $F_{y}$ are the main and radial cutting forces, β is friction angle, and $\gamma_{0}$ is rake angle, respectively. From Figure 4c,d, it can be concluded that β’ < β, as both tools are used under equivalent situations during the turning of Inconel 718. Thus, if we assume that $F_{n}$ remains constant, Equations (1)–(3) show that the cutting forces decreased using Tool 2. The cutting force is an important factor that affects the cutting temperature. Therefore, the microgroove structure changed the balance of the cutting force, led to a decrease in contact friction between the tool and chip, and thus the cutting temperature of Tool 2 decreased.

### 3.2. Effect of Microgroove on Tool Wear of Rake and Flank Face

**Figure 5:**
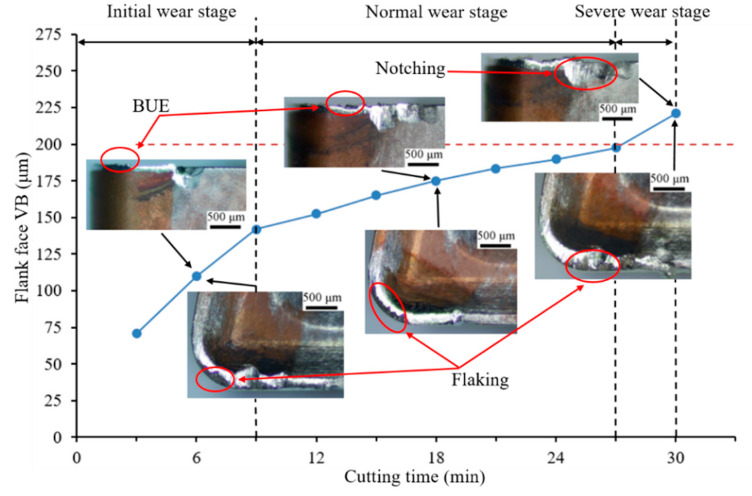
Tool wear evolution of Tool 1 on rake and flank faces ($v_{c}$: 35 m/min, $a_{p}$: 1.5 mm, *f*: 0.1 mm/r).

**Figure 6:**
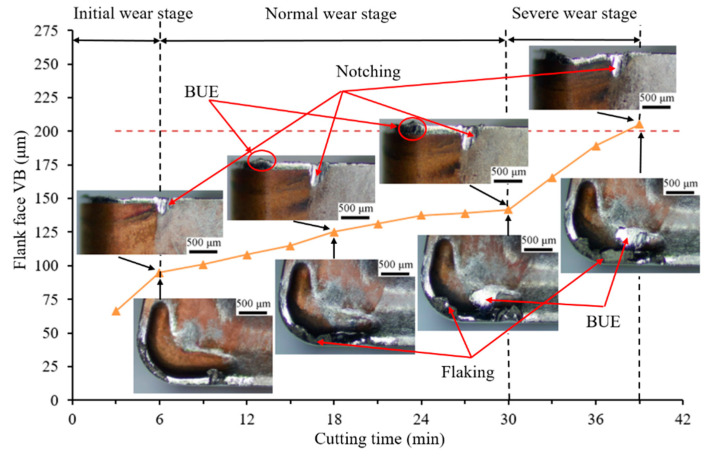
Tool wear evolution of Tool 2 on rake and flank faces ($v_{c}$: 35 m/min, $a_{p}$: 1.5 mm, f: 0.1 mm/r).

The flank wear processes that were achieved for Tools 1 and 2 in the overall turning period are shown in Figure 5 and Figure 6, respectively, and provide a tool life comparison between Tools 1 and 2 cutting. When Inconel 718 was turned for 30 and 39 min, Tools 1 and 2 reached maximum wear, respectively. The wear on the rake and flank faces of the tools as studied by optical microscopy confirmed that the tool life of Tool 2 outperformed that of Tool 1 to a maximum of a 23.08% longer tool life.

Figure 5 and Figure 6 show that the wear of Tool 1 is more severe than that of Tool 2 on the rake and flank faces during the equivalent machining period. Both cases underwent three stages of wear: initial, normal, and severe wear. The normal wear stage of Tool 2 was longer, and the change in wear VB was not obvious. The wear curve of Tool 1 was steeper than that of Tool 2 from 9 to 27 min, which indicates that the wear of Tool 1 was rapid.

The wear rate of Tool 1 was faster, and the flank wear was irregular from a comparison of the flank wear VB of the two tools. After 6 min of cutting, a slight chipping was observed near the DOC (depth of cut) line on the flank face. With continuous cutting, the coating on the rake face of Tool 1 was peeled off. The substrate material was exposed gradually, which increased the cutting forces and temperature, and the tool wear was accelerated during the remainder of the test. Therefore, the chipping of Tool 1 was more obvious when cutting for 18 and 30 min. However, the flank wear was relatively uniform and regular for Tool 2, and slight notch wear resulted near the DOC line on the flank face. When cutting for 30 min, the flank wear of Tool 1 exceeded 200 μm (VB wear standard), whereas the cutting edge of Tool 2 retained a good integrity and no chipping resulted. The main cutting edge of Tool 2 was not damaged until 39 min. The bottom of the microgroove was not worn, as shown in Figure 6, which indicates that the bottom of the chip and the microgroove were not in full contact. This result is consistent with the simulation results in Section 3.1.

We concluded that Tool 2 exhibits a better wear resistance performance. The main reason for this behavior is that the microgroove structure changes the contact form of the tool–chip, which changes the force balance during cutting, and leads to the decrease in cutting force and temperature. The cutting force and temperature are significant factors that affect tool life. Thus, the tool life of Tool 2 is longer than that of Tool 1.

### 3.3. Effect of Microgroove on Chip Morphology

#### 3.3.1. Chip Curl Radius

During cutting, chips usually curl and then break naturally, or flow out as a band fracture [34]. However, some chips break into small pieces and eject directly, which poses a threat to personal safety, and reduces the surface quality of the workpiece. The chip morphology and curl radius of Tool 1 and Tool 2 were compared, and the effect of microgroove structure on chip deformation was studied.

**Figure 7:**
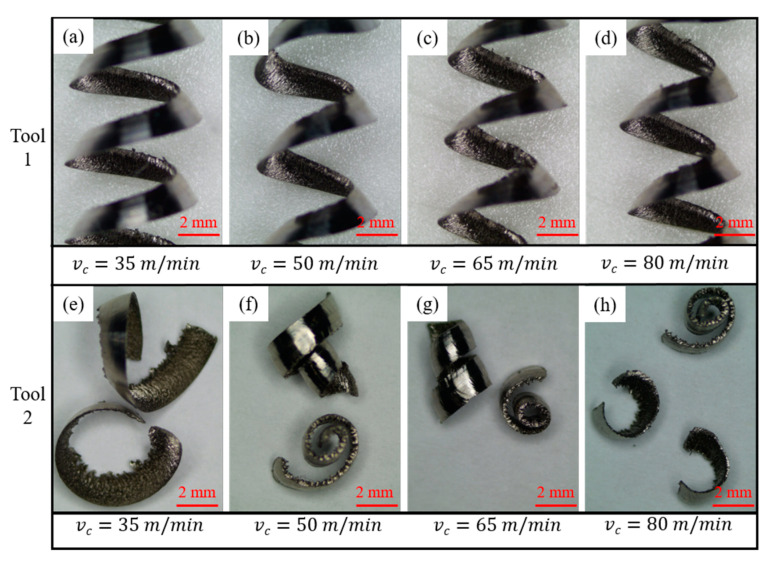
Comparison of chip shape and curl radius after cutting with two tools: (**a**–**d**) the chip shape and curl radius of Tool 1 at cutting speeds of 35, 50, 65 and 80 m/min, respectively, (**e**–**h**) the chip shape and curl radius of Tool 2 at cutting speeds of 35, 50, 65 and 80 m/min, respectively.

The results for Tools 1 and 2 in the four cutting tests in Section 2.1 agreed for the same cutting parameters and conditions. After the cutting test from each group, the chips were collected, and the shape and curl radius of the chips were observed by optical microscopy after ultrasonic cleaning. The results are shown in Figure 7.

Figure 7 shows that when Tool 1 was used, the chips that were generated at four different cutting speeds were of a long continuous spring-type, and the curl radius of each was close. The cutting speed influences the chip shape and curl radius for Tool 2. With an increase in cutting speed, the curl radius of these chips decreased gradually. As shown in Figure 7e–h, the chip morphology included three main types: long ear-type, conical helical, and C-type chips.

**Figure 8:**
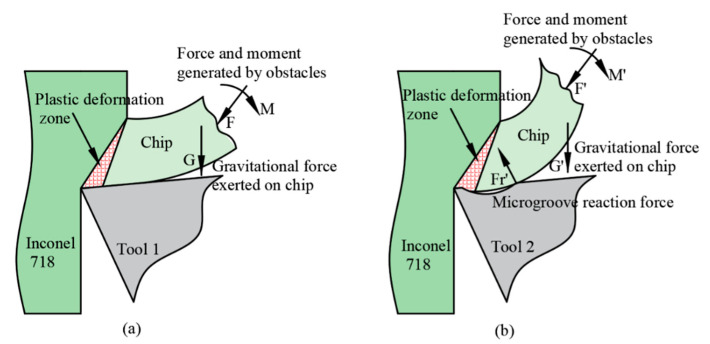
Deformation analysis of chips during the turning of Inconel 718 for both tools: (**a**) the chip deformation analysis for Tool 1, (**b**) the chip deformation analysis for Tool 2.

An analysis of the turning of superalloy Inconel 718 indicates that the workpiece material at the root of the chip underwent severe plastic deformation to form chips, and the superalloy material existed in a yield state at this time. The high temperature was caused by the plastic deformation of the superalloy, which caused the material to soften and promoted plastic flow [35]. In the case of plastic flow, a small change in stress distribution can cause a large change in strain. If a small external force is applied to the plastic deformation zone, the shear slip can be affected significantly. Thus, we performed a force analysis on both cutting tools, and the comparison is shown in Figure 8.

Figure 8a shows the contact state of the tool–chip and chip deformation for Tool 1. Chips with a larger curl radius were produced along the rake face of the tool and flowed out of the deformation area in a straight continuous ribbon shape, which was difficult to break. When the cutting speed increased, the chip bent sideways to form a long continuous conical helical chip. When the plastic deformation exceeded the ultimate strain of material, the chip broke naturally. Figure 8b shows the contact state of tool–chip and chip deformation of Tool 2. The chip flowed from the front to the end along the microgroove and was subjected to a reaction force $F_{r}\prime$ at the end edge of the microgroove, which changed the stress distribution at the root of the chip and the chip shape. During the cutting of Inconel 718, the effects of various obstacles, such as workpiece, tool bar, machine tool, and chip gravity, and external forces produce a force and moment at the root of chip, which leads to a change in stress distribution. At this time, the shear slip state in the plastic zone must change rapidly to adapt to the new stress state. The main method is to reconstruct the balance of force and moment on the chip by using the shape change of the shear plane. Subsequently, the shape change of the shear plane leads to a change in chip shape [36]. Therefore, when Inconel 718 was turned with Tool 2, the chip deformed for the first time, and a second deformation occurred when the chip flowed through the end of the microgroove. As a result, the curl radius of the chips was reduced, and the chip breaking performance was improved.

To achieve an innovative tool microgroove design, most literature has focused on the design and optimization of chip breaking and anti-wear grooves. Lotfi et al. [37] studied the influence of geometric parameters of chip breakers on the main deformation area, cutting force, and chip deformation, and found that chip breakers with a larger rake angle and deeper microgroove depth can reduce the cutting force, as well as have a good effect on chip deformation and chip breaking. Bahattin et al. [38] developed an external chip breaker-assisted turning operation system. Results showed that the system exhibited excellent chip breakability: the chip breaker reduced cutting forces and temperatures, and the surface quality improved. However, the external system affected the tool-changing operation and reduced the cutting efficiency. It is noted that the microgroove structure designed in this paper was smaller than the chip breaker and could therefore improve the tool life and chip breaking effect. Further, the microgroove tool was created using a powder metallurgy compaction process, which does not cause thermal ablation during laser processing like micro-textured tools.

#### 3.3.2. Chip Burr

**Figure 9:**
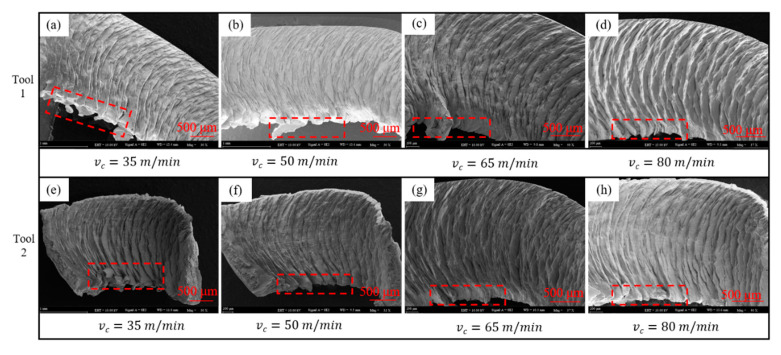
Comparison of chip burr after turning with Tool 1 and Tool 2: (**a**–**d**) the chip burr of Tool 1 at cutting speeds of 35, 50, 65 and 80 m/min, re-spectively, (**e**–**h**) the chip burr of Tool 2 at cutting speeds of 35, 50, 65 and 80 m/min, respectively.

The chips that were collected from each group were cleaned with acetone solution and dried rapidly. The microscopic details and EDS analysis of the chips were obtained by a scanning electron microscope. SEM analysis of the chips showed that chip burrs were clearly visible in each group, as shown in Figure 9.

Figure 9 shows varying degrees of burrs at the lower edge of the chips, whereas the upper edge of the chips was relatively smooth at different cutting speeds, particularly for chips that were obtained by processing with the two tools. The chip burr decreased with an increase in cutting speed, and the chip burr phenomenon caused by Tool 2 was not as serious as that of Tool 1. When the cutting speed was relatively low (i.e., $v_{c}$ = 35 m/min), the serrated burr was clearly visible on the lower side of the chip that was generated by Tool 1, with a large size and dense distribution. However, scattered and small serrated burrs existed on the lower edge of the chip that was produced by Tool 2. When the cutting speed increased, fewer burrs were produced on the chips from the two cutting tools, and the degree of the burr was closer. These results indicate that the chip burr decreased with an increase in cutting speed, and when the cutting speed was low, the size of the chip burr was large, and the distribution was concentrated.

## 4. Conclusions

This study investigated the effect of microgroove structure on cutting performance and chip morphology during the turning of superalloy Inconel 718. The results from this work establish the usefulness of microgroove cutting tools to improve tool life and chip breaking. After a detailed analysis of the results, the following conclusions were reached:

(1) The cutting force and temperature of the microgroove cutting tool were reduced for the recommended cutting parameters. The tool life of the microgroove cutting tool was 23.08% longer than that of the original cutting tool, mainly because the microgroove structure improves the contact friction at the tool–chip interface.
(2) Because of the microgroove structure, the chip curl radius was reduced, and the breaking performance of the chip was improved.
(3) The chip burrs decreased with an increase in cutting speed, and the chip burr phenomenon that was caused by the microgroove cutting tool was not as intense as that of the original cutting tool.
(4) Microgroove cutting tool is more suitable for machining superalloy Inconel 718, due to its high durability and good effect of chip breaking.

## Figures and Tables

**Table 1:** Input process parameters of turning superalloy Inconel 718.

Cutting Tool	Original Cutting Tool (Tool 1) Microgroove Cutting Tool (Tool 2)
Workpiece material	Superalloy Inconel 718
	Ni 51.80%, Fe 20.58%, Cr 17.20%, Nb 4.90%
	Mo 3.40%, Ti 0.80%, Al 0.40%, C 0.02%
Workpiece dimension	ϕ60×230 mm
Cutting speed	$v_{c}=35,50,65,80\;m/\min$
Feed rate	f=0.1 mm/rev
Depth of cut	$a_{p}=1.5\;\mathrm{mm}$
Response considered	Cutting force, cutting temperature, tool wear, and chip morphology

**Table 2:** Physical properties of superalloy Inconel 718.

Density ρ g/cm^3^	Hardness (HBS)	Poisson Ratio μ	Elasticity Modulus E (GPa)	$\mathrm{Yield}\;\mathrm{Strength}\;\sigma_{s}(\mathrm{MPa})$	$\mathrm{Tensile}\;\mathrm{Strength}\;\sigma_{b}(\mathrm{MPa})$
8.24	363	0.3	199.9	550	965

**Table 3:** Finite-element simulation condition settings.

Object	Tool	Workpiece
Material	WC	Inconel 718
Material type	Rigid	Plastic
Element type	Tetrahedral	Tetrahedral
Number of elements	155,669	45,987
Cutting parameters	$v_{c}$ = 35 m/min, $a_{p}$ = 1.5 mm, f = 0.1 mm/rev	
Primary environmental temperature	20 °C	
Shear friction factor	0.4	
Heat transfer coefficient	2000 N/s/mm/C	
Cutting length	15.7 mm	
Size ratio	0.7	

## Data Availability

The raw/processed data required to reproduce these findings cannot be shared at this time as the data also form part of an ongoing study.

## Abbreviations

WC	wolfram carbide
MQL	minimum quantity lubrication
BUE	built up edge
PVD	physical vapor deposition
